# Supplementary material for: What empowerment indicators are important for food consumption for women? Evidence from 5 sub-Sahara African countries
Source: PLoS One. 2021 Apr 21;16(4):e0250014. doi: 10.1371/journal.pone.0250014 (PMC8059862; doi:10.1371/journal.pone.0250014)
Supplement: S1 Table — (DOCX) [file pone.0250014.s001.docx]

S1 Table. Marginal effects of Poisson regression for WDDS – Empowerment score

|  | (1) | (2) | (3) | (4) | (5) | (6) |
| --- | --- | --- | --- | --- | --- | --- |
| VARIABLES | All | Mozambique | Rwanda | Malawi | Uganda | Zambia |
| Empower score | 0.092 | 0.685** | 1.333*** | -0.004 | -0.483 | -0.087 |
|  | (0.204) | (0.340) | (0.374) | (0.220) | (0.348) | (0.258) |
| SES index | -0.043 | 0.035 | 0.567 | 0.273** | -0.911** | -1.846** |
|  | (0.107) | (0.330) | (1.050) | (0.125) | (0.406) | (0.736) |
| SES index squared | 0.020 | 0.130 | 0.179 | 0.019 | 0.144*** | -1.038** |
|  | (0.015) | (0.214) | (0.345) | (0.015) | (0.055) | (0.438) |
| Men’s age | 0.005*** | 0.007* | 0.002 | 0.006* | 0.008*** | 0.003 |
|  | (0.002) | (0.004) | (0.002) | (0.003) | (0.003) | (0.003) |
| Women’s age | -0.012*** | -0.009** | -0.010*** | -0.015*** | -0.013*** | -0.003 |
|  | (0.002) | (0.004) | (0.004) | (0.003) | (0.004) | (0.003) |
| Women’s education | 0.041*** | 0.061 | 0.100*** | 0.081** | 0.032*** | 0.040*** |
|  | (0.009) | (0.060) | (0.032) | (0.037) | (0.010) | (0.013) |
| Household size | 0.040*** | 0.045* | 0.046 | 0.044** | 0.025 | 0.044*** |
|  | (0.012) | (0.026) | (0.034) | (0.022) | (0.017) | (0.012) |
| Study location | -0.014** | 0.075*** | 0.022*** | 0.026 | -0.028*** | -0.078 |
|  | (0.006) | (0.016) | (0.008) | (0.057) | (0.007) | (0.073) |
| Study month^a^ | |  |  |  |  |  |
| February | 0.053 | 0.154 |  |  |  |  |
|  | (0.253) | (0.121) |  |  |  |  |
| March | -0.592*** | -0.297* |  |  |  |  |
|  | (0.181) | (0.178) |  |  |  |  |
| April | -0.253 | 0.565* |  |  |  |  |
|  | (0.220) | (0.343) |  |  |  |  |
| November | -0.001 | 0.347** |  | -2.430*** | 0.518 |  |
|  | (0.159) | (0.142) |  | (0.231) | (0.324) |  |
| December | 0.154 | -0.330** | 0.298** | -2.325*** | -0.058 | -0.044 |
|  | (0.126) | (0.141) | (0.116) | (0.380) | (0.204) | (0.216) |
| Countries [*Ref: Mozambique*] | |  |  |  |  |  |
| Malawi | -0.219 |  |  |  |  |  |
|  | (0.213) |  |  |  |  |  |
| Rwanda | -0.267 |  |  |  |  |  |
|  | (0.181) |  |  |  |  |  |
| Uganda | -0.736* |  |  |  |  |  |
|  | (0.376) |  |  |  |  |  |
| Zambia | -0.003 |  |  |  |  |  |
|  | (0.177) |  |  |  |  |  |
| Observations | 18,117 | 2,100 | 3,681 | 4,569 | 3,754 | 4,013 |

Note: Standard errors in parentheses; *** p<0.01, ** p<0.05, * p<0.1; ^a^Ref categories; January (Pooled, Mozambique, Rwanda, Malawi, Uganda), November (Zambia)
