# Supplementary material for: What empowerment indicators are important for food consumption for women? Evidence from 5 sub-Sahara African countries
Source: PLoS One. 2021 Apr 21;16(4):e0250014. doi: 10.1371/journal.pone.0250014 (PMC8059862; doi:10.1371/journal.pone.0250014)
Supplement: S2 Table — (DOCX) [file pone.0250014.s002.docx]

S2 Table. Marginal effects of Poisson regression for WDDS – Prod domain (Aut in production)

|  | (1) | (2) | (3) | (4) | (5) | (6) |
| --- | --- | --- | --- | --- | --- | --- |
| VARIABLES | All | Mozambique | Rwanda | Malawi | Uganda | Zambia |
| Aut in production decs | 0.181** | 0.054 | 0.108 | 0.081 | 0.333** | 0.101 |
|  | (0.072) | (0.134) | (0.111) | (0.066) | (0.146) | (0.086) |
| SES index | -0.016 | 0.013 | 0.665 | -0.293** | -0.664 | -1.746** |
|  | (0.107) | (0.317) | (1.002) | (0.129) | (0.540) | (0.698) |
| SES index squared | 0.018 | 0.124 | 0.207 | 0.021 | 0.113 | -0.967** |
|  | (0.014) | (0.214) | (0.330) | (0.015) | (0.072) | (0.420) |
| Men’s age | 0.005*** | 0.007* | 0.003 | 0.006* | 0.008*** | 0.003 |
|  | (0.001) | (0.004) | (0.002) | (0.003) | (0.002) | (0.003) |
| Women’s age | -0.011*** | -0.012*** | -0.010*** | -0.015*** | -0.012*** | -0.003 |
|  | (0.002) | (0.004) | (0.004) | (0.003) | (0.003) | (0.003) |
| Women’s education | 0.042*** | 0.009 | 0.118*** | 0.084** | 0.031*** | 0.040*** |
|  | (0.009) | (0.061) | (0.030) | (0.037) | (0.010) | (0.013) |
| Household size | 0.032** | 0.048* | 0.050 | 0.035* | 0.014 | 0.042*** |
|  | (0.013) | (0.026) | (0.032) | (0.020) | (0.018) | (0.012) |
| Study location | -0.015*** | 0.061*** | 0.018** | 0.020 | -0.028*** | -0.069 |
|  | (0.005) | (0.013) | (0.008) | (0.056) | (0.006) | (0.071) |
| Study month^a^ |  |  |  |  |  |  |
| February | 0.086 | 0.013 |  |  |  |  |
|  | (0.234) | (0.132) |  |  |  |  |
| March | -0.621*** | -0.417** |  |  |  |  |
|  | (0.179) | (0.178) |  |  |  |  |
| April | -0.163 | 0.445 |  |  |  |  |
|  | (0.213) | (0.275) |  |  |  |  |
| November | 0.023 | 0.349** |  | -2.399*** | 0.390 |  |
|  | (0.153) | (0.136) |  | (0.221) | (0.328) |  |
| December | 0.184 | -0.381** | 0.301*** | -2.261*** | -0.193 | -0.052 |
|  | (0.119) | (0.161) | (0.113) | (0.369) | (0.254) | (0.216) |
| Countries [*Ref: Mozambique*] | |  |  |  |  |  |
| Malawi | -0.210 |  |  |  |  |  |
|  | (0.221) |  |  |  |  |  |
| Rwanda | -0.306* |  |  |  |  |  |
|  | (0.183) |  |  |  |  |  |
| Uganda | -0.830** |  |  |  |  |  |
|  | (0.378) |  |  |  |  |  |
| Zambia | -0.018 |  |  |  |  |  |
|  | (0.180) |  |  |  |  |  |
| Observations | 19,668 | 2,594 | 4,011 | 4,773 | 4,063 | 4,227 |

Note: Standard errors in parentheses; *** p<0.01, ** p<0.05, * p<0.1; ^a^Ref categories; January (Pooled, Mozambique, Rwanda, Malawi, Uganda), November (Zambia)
