# Supplementary material for: What empowerment indicators are important for food consumption for women? Evidence from 5 sub-Sahara African countries
Source: PLoS One. 2021 Apr 21;16(4):e0250014. doi: 10.1371/journal.pone.0250014 (PMC8059862; doi:10.1371/journal.pone.0250014)
Supplement: S3 Table — (DOCX) [file pone.0250014.s003.docx]

S3 Table. Marginal effects of Poisson regression for WDDS – Prod domain (Input in productive decisions)

|  | (1) | (2) | (3) | (4) | (5) | (6) |
| --- | --- | --- | --- | --- | --- | --- |
| VARIABLES | All | Mozambique | Rwanda | Malawi | Uganda | Zambia |
| Input in productive decs | 0.222*** | -0.070 | 0.543*** | 0.051 | 0.132 | 0.184* |
|  | (0.058) | (0.084) | (0.103) | (0.081) | (0.096) | (0.102) |
| SES index | -0.016 | 0.023 | 0.902 | -0.281** | -0.693 | -1.670** |
|  | (0.108) | (0.319) | (1.009) | (0.129) | (0.533) | (0.676) |
| SES index squared | 0.019 | 0.137 | 0.279 | 0.019 | 0.118* | -0.910** |
|  | (0.014) | (0.217) | (0.334) | (0.015) | (0.071) | (0.405) |
| Men’s age | 0.005*** | 0.006 | 0.002 | 0.005* | 0.008*** | 0.003 |
|  | (0.001) | (0.004) | (0.002) | (0.003) | (0.003) | (0.003) |
| Women’s age | -0.012*** | -0.011*** | -0.010*** | -0.016*** | -0.013*** | -0.003 |
|  | (0.002) | (0.004) | (0.004) | (0.003) | (0.004) | (0.003) |
| Women’s education | 0.038*** | -0.012 | 0.106*** | 0.079** | 0.028*** | 0.039*** |
|  | (0.010) | (0.070) | (0.032) | (0.037) | (0.011) | (0.012) |
| Household size | 0.029** | 0.051** | 0.032 | 0.034* | 0.012 | 0.043*** |
|  | (0.013) | (0.025) | (0.030) | (0.021) | (0.019) | (0.011) |
| Study location | -0.015*** | 0.062*** | 0.020** | 0.019 | -0.029*** | -0.070 |
|  | (0.005) | (0.014) | (0.008) | (0.056) | (0.007) | (0.070) |
| Study month^a^ |  |  |  |  |  |  |
| February | 0.111 | -0.022 |  |  |  |  |
|  | (0.237) | (0.118) |  |  |  |  |
| March | -0.593*** | -0.452** |  |  |  |  |
|  | (0.184) | (0.178) |  |  |  |  |
| April | -0.178 | 0.395 |  |  |  |  |
|  | (0.226) | (0.300) |  |  |  |  |
| November | -0.023 | 0.280** |  | -2.402*** | 0.405 |  |
|  | (0.156) | (0.130) |  | (0.219) | (0.303) |  |
| December | 0.134 | -0.420*** | 0.240** | -2.267*** | -0.231 | -0.043 |
|  | (0.123) | (0.153) | (0.113) | (0.369) | (0.227) | (0.211) |
| Countries [*Ref: Mozambique*] | |  |  |  |  |  |
| Malawi | -0.234 |  |  |  |  |  |
|  | (0.216) |  |  |  |  |  |
| Rwanda | -0.328* |  |  |  |  |  |
|  | (0.185) |  |  |  |  |  |
| Uganda | -0.850** |  |  |  |  |  |
|  | (0.384) |  |  |  |  |  |
| Zambia | -0.056 |  |  |  |  |  |
|  | (0.182) |  |  |  |  |  |
| Observations | 19,216 | 2,558 | 3,832 | 4,657 | 3,947 | 4,222 |

Note: Standard errors in parentheses; *** p<0.01, ** p<0.05, * p<0.1; ^a^Ref categories; January (Pooled, Mozambique, Rwanda, Malawi, Uganda), November (Zambia)
