# Supplementary material for: What empowerment indicators are important for food consumption for women? Evidence from 5 sub-Sahara African countries
Source: PLoS One. 2021 Apr 21;16(4):e0250014. doi: 10.1371/journal.pone.0250014 (PMC8059862; doi:10.1371/journal.pone.0250014)
Supplement: S4 Table — (DOCX) [file pone.0250014.s004.docx]

S4 Table. Marginal effects of Poisson regression results for WDDS – Resources domains (Asset ownership)

|  | (1) | (2) | (3) | (4) | (5) | (6) |
| --- | --- | --- | --- | --- | --- | --- |
| VARIABLES | All | Mozambique | Rwanda | Malawi | Uganda | Zambia |
| Sole/Joint ownrshp asset | 0.056 | 0.399* | 0.401** | 0.117 | 0.016 | -0.214* |
|  | (0.099) | (0.229) | (0.195) | (0.111) | (0.154) | (0.125) |
| SES index | -0.014 | 0.028 | 0.646 | -0.290** | -0.656 | -1.689** |
|  | (0.107) | (0.311) | (0.991) | (0.131) | (0.546) | (0.684) |
| SES index squared | 0.018 | 0.123 | 0.201 | 0.020 | 0.112 | -0.944** |
|  | (0.014) | (0.210) | (0.327) | (0.015) | (0.073) | (0.412) |
| Men’s age | 0.005*** | 0.007* | 0.003 | 0.005* | 0.008*** | 0.003 |
|  | (0.001) | (0.004) | (0.002) | (0.003) | (0.003) | (0.003) |
| Women’s age | -0.011*** | -0.012*** | -0.010*** | -0.016*** | -0.012*** | -0.003 |
|  | (0.002) | (0.004) | (0.004) | (0.003) | (0.003) | (0.003) |
| Women’s education | 0.042*** | 0.015 | 0.119*** | 0.084** | 0.032*** | 0.042*** |
|  | (0.009) | (0.060) | (0.030) | (0.036) | (0.010) | (0.012) |
| Household size | 0.032** | 0.048* | 0.048 | 0.035* | 0.013 | 0.044*** |
|  | (0.013) | (0.025) | (0.032) | (0.020) | (0.019) | (0.011) |
| Study location | -0.014*** | 0.061*** | 0.018** | 0.017 | -0.027*** | -0.075 |
|  | (0.005) | (0.014) | (0.008) | (0.056) | (0.006) | (0.070) |
| Study month^a^ |  |  |  |  |  |  |
| February | 0.110 | 0.013 |  |  |  |  |
|  | (0.235) | (0.112) |  |  |  |  |
| March | -0.601*** | -0.421** |  |  |  |  |
|  | (0.182) | (0.179) |  |  |  |  |
| April | -0.159 | 0.424 |  |  |  |  |
|  | (0.214) | (0.287) |  |  |  |  |
| November | 0.027 | 0.336*** |  | -2.389*** | 0.379 |  |
|  | (0.154) | (0.125) |  | (0.220) | (0.344) |  |
| December | 0.179 | -0.401*** | 0.306*** | -2.247*** | -0.156 | -0.074 |
|  | (0.120) | (0.148) | (0.113) | (0.369) | (0.282) | (0.209) |
| Countries [*Ref: Mozambique*] | |  |  |  |  |  |
| Malawi | -0.202 |  |  |  |  |  |
|  | (0.221) |  |  |  |  |  |
| Rwanda | -0.273 |  |  |  |  |  |
|  | (0.183) |  |  |  |  |  |
| Uganda | -0.829** |  |  |  |  |  |
|  | (0.376) |  |  |  |  |  |
| Zambia | -0.003 |  |  |  |  |  |
|  | (0.179) |  |  |  |  |  |
| Observations | 19,709 | 2,594 | 4,031 | 4,777 | 4,068 | 4,239 |

Note: Standard errors in parentheses; *** p<0.01, ** p<0.05, * p<0.1; ^a^Ref categories; January (Pooled, Mozambique, Rwanda, Malawi, Uganda), November (Zambia)
