# Supplementary material for: What empowerment indicators are important for food consumption for women? Evidence from 5 sub-Sahara African countries
Source: PLoS One. 2021 Apr 21;16(4):e0250014. doi: 10.1371/journal.pone.0250014 (PMC8059862; doi:10.1371/journal.pone.0250014)
Supplement: S5 Table — (DOCX) [file pone.0250014.s005.docx]

S5 Table. Marginal effects of Poisson regression results for WDDS – Resources domain (asset sale, purchase, and transfer)

|  | (1) | (2) | (3) | (4) | (5) | (6) |
| --- | --- | --- | --- | --- | --- | --- |
| VARIABLES | All | Mozambique | Rwanda | Malawi | Uganda | Zambia |
| Input in asset transactions | 0.048 | 0.324** | 0.481*** | -0.102 | -0.153 | -0.121 |
|  | (0.076) | (0.134) | (0.127) | (0.108) | (0.130) | (0.092) |
| SES index | -0.014 | 0.006 | 0.770 | -0.291** | -0.668 | -1.778** |
|  | (0.107) | (0.311) | (1.025) | (0.130) | (0.543) | (0.694) |
| SES index squared | 0.018 | 0.113 | 0.239 | 0.021 | 0.114 | -0.996** |
|  | (0.014) | (0.209) | (0.336) | (0.015) | (0.073) | (0.417) |
| Men’s age | 0.005*** | 0.007* | 0.002 | 0.006* | 0.009*** | 0.003 |
|  | (0.001) | (0.004) | (0.002) | (0.003) | (0.003) | (0.003) |
| Women’s age | -0.011*** | -0.013*** | -0.010*** | -0.015*** | -0.012*** | -0.003 |
|  | (0.002) | (0.004) | (0.004) | (0.003) | (0.003) | (0.003) |
| Women’s education | 0.042*** | 0.018 | 0.122*** | 0.084** | 0.033*** | 0.040*** |
|  | (0.009) | (0.061) | (0.031) | (0.036) | (0.010) | (0.013) |
| Household size | 0.032** | 0.045* | 0.041 | 0.035* | 0.014 | 0.044*** |
|  | (0.013) | (0.025) | (0.031) | (0.019) | (0.018) | (0.012) |
| Study location | -0.014*** | 0.061*** | 0.018** | 0.021 | -0.028*** | -0.073 |
|  | (0.005) | (0.013) | (0.008) | (0.056) | (0.006) | (0.071) |
| Study month^a^ |  |  |  |  |  |  |
| February | 0.107 | 0.024 |  |  |  |  |
|  | (0.235) | (0.112) |  |  |  |  |
| March | -0.604*** | -0.423** |  |  |  |  |
|  | (0.182) | (0.178) |  |  |  |  |
| April | -0.159 | 0.442 |  |  |  |  |
|  | (0.215) | (0.289) |  |  |  |  |
| November | 0.025 | 0.360*** |  | -2.421*** | 0.537 |  |
|  | (0.155) | (0.131) |  | (0.227) | (0.362) |  |
| December | 0.177 | -0.392*** | 0.290*** | -2.293*** | -0.033 | -0.065 |
|  | (0.121) | (0.147) | (0.110) | (0.377) | (0.294) | (0.215) |
| Countries [*Ref: Mozambique*] | |  |  |  |  |  |
| Malawi | -0.200 |  |  |  |  |  |
|  | (0.221) |  |  |  |  |  |
| Rwanda | -0.268 |  |  |  |  |  |
|  | (0.183) |  |  |  |  |  |
| Uganda | -0.831** |  |  |  |  |  |
|  | (0.376) |  |  |  |  |  |
| Zambia | 0.000 |  |  |  |  |  |
|  | (0.180) |  |  |  |  |  |
| Observations | 19,709 | 2,594 | 4,031 | 4,777 | 4,068 | 4,239 |

Note: Standard errors in parentheses; *** p<0.01, ** p<0.05, * p<0.1; ^a^Ref categories; January (Pooled, Mozambique, Rwanda, Malawi, Uganda), November (Zambia)
