# Supplementary material for: What empowerment indicators are important for food consumption for women? Evidence from 5 sub-Sahara African countries
Source: PLoS One. 2021 Apr 21;16(4):e0250014. doi: 10.1371/journal.pone.0250014 (PMC8059862; doi:10.1371/journal.pone.0250014)
Supplement: S6 Table — (DOCX) [file pone.0250014.s006.docx]

S6 Table. Marginal effects of Poisson regression results for WDDS – Resources domain (input in ≥ 1 credit source)

|  | (1) | (2) | (3) | (4) | (5) | (6) |
| --- | --- | --- | --- | --- | --- | --- |
| VARIABLES | All | Mozambique | Rwanda | Malawi | Uganda | Zambia |
| Input in credit source decs | -0.040 | 0.207 | 0.250*** | -0.089 | -0.196** | -0.056 |
|  | (0.056) | (0.185) | (0.070) | (0.077) | (0.093) | (0.062) |
| SES index | -0.005 | 0.032 | 0.599 | -0.292** | -0.675 | -1.781** |
|  | (0.107) | (0.317) | (0.984) | (0.130) | (0.531) | (0.701) |
| SES index squared | 0.017 | 0.131 | 0.190 | 0.021 | 0.115 | -1.007** |
|  | (0.014) | (0.216) | (0.325) | (0.015) | (0.071) | (0.420) |
| Men’s age | 0.006*** | 0.007** | 0.003 | 0.006* | 0.009*** | 0.003 |
|  | (0.001) | (0.004) | (0.002) | (0.003) | (0.003) | (0.003) |
| Women’s age | -0.011*** | -0.012*** | -0.010** | -0.016*** | -0.011*** | -0.003 |
|  | (0.002) | (0.004) | (0.004) | (0.003) | (0.004) | (0.003) |
| Women’s education | 0.043*** | 0.011 | 0.122*** | 0.084** | 0.035*** | 0.041*** |
|  | (0.009) | (0.061) | (0.030) | (0.037) | (0.010) | (0.013) |
| Household size | 0.032** | 0.045* | 0.042 | 0.035* | 0.014 | 0.044*** |
|  | (0.013) | (0.026) | (0.031) | (0.020) | (0.018) | (0.012) |
| Study location | -0.015*** | 0.062*** | 0.020** | 0.019 | -0.028*** | -0.077 |
|  | (0.005) | (0.013) | (0.008) | (0.055) | (0.006) | (0.072) |
| Study month^a^ |  |  |  |  |  |  |
| February | 0.084 | -0.046 |  |  |  |  |
|  | (0.235) | (0.121) |  |  |  |  |
| March | -0.614*** | -0.465** |  |  |  |  |
|  | (0.182) | (0.183) |  |  |  |  |
| April | -0.168 | 0.410 |  |  |  |  |
|  | (0.214) | (0.289) |  |  |  |  |
| November | 0.015 | 0.314** |  | -2.411*** | 0.464 |  |
|  | (0.154) | (0.129) |  | (0.220) | (0.356) |  |
| December | 0.174 | -0.415*** | 0.323*** | -2.271*** | -0.084 | -0.048 |
|  | (0.121) | (0.149) | (0.109) | (0.368) | (0.306) | (0.217) |
| Countries [*Ref: Mozambique*] | |  |  |  |  |  |
| Malawi | -0.176 |  |  |  |  |  |
|  | (0.223) |  |  |  |  |  |
| Rwanda | -0.267 |  |  |  |  |  |
|  | (0.183) |  |  |  |  |  |
| Uganda | -0.846** |  |  |  |  |  |
|  | (0.380) |  |  |  |  |  |
| Zambia | -0.005 |  |  |  |  |  |
|  | (0.179) |  |  |  |  |  |
| Observations | 19,576 | 2,559 | 4,013 | 4,746 | 4,052 | 4,206 |

Note: Standard errors in parentheses; *** p<0.01, ** p<0.05, * p<0.1; ^a^Ref categories; January (Pooled, Mozambique, Rwanda, Malawi, Uganda), November (Zambia)
