# Supplementary material for: What empowerment indicators are important for food consumption for women? Evidence from 5 sub-Sahara African countries
Source: PLoS One. 2021 Apr 21;16(4):e0250014. doi: 10.1371/journal.pone.0250014 (PMC8059862; doi:10.1371/journal.pone.0250014)
Supplement: S7 Table — (DOCX) [file pone.0250014.s007.docx]

S7 Table. Marginal effects of Poisson regression results for WDDS – Income domain (Input in at least 1 income decision)

|  | (1) | (2) | (3) | (4) | (5) | (6) |
| --- | --- | --- | --- | --- | --- | --- |
| VARIABLES | All | Mozambique | Rwanda | Malawi | Uganda | Zambia |
| Input in ≥1 income domain | -0.037 | -0.040 | 0.096 | 0.199 | -0.121 | -0.063 |
|  | (0.112) | (0.140) | (0.233) | (0.149) | (0.174) | (0.118) |
| SES index | -0.017 | 0.018 | 0.671 | -0.293** | -0.693 | -1.772** |
|  | (0.108) | (0.320) | (1.025) | (0.132) | (0.547) | (0.695) |
| SES index squared | 0.019 | 0.129 | 0.212 | 0.021 | 0.118 | -0.989** |
|  | (0.014) | (0.217) | (0.337) | (0.015) | (0.073) | (0.417) |
| Men’s age | 0.005*** | 0.007* | 0.003 | 0.005* | 0.008*** | 0.003 |
|  | (0.001) | (0.004) | (0.002) | (0.003) | (0.003) | (0.003) |
| Women’s age | -0.011*** | -0.012*** | -0.010*** | -0.016*** | -0.012*** | -0.003 |
|  | (0.002) | (0.004) | (0.004) | (0.003) | (0.004) | (0.003) |
| Women’s education | 0.043*** | 0.009 | 0.122*** | 0.084** | 0.032*** | 0.040*** |
|  | (0.010) | (0.062) | (0.031) | (0.036) | (0.011) | (0.013) |
| Household size | 0.032** | 0.048* | 0.048 | 0.034* | 0.013 | 0.044*** |
|  | (0.013) | (0.026) | (0.032) | (0.020) | (0.019) | (0.012) |
| Study location | -0.014*** | 0.061*** | 0.018** | 0.024 | -0.027*** | -0.075 |
|  | (0.005) | (0.014) | (0.008) | (0.056) | (0.006) | (0.072) |
| Study month^a^ |  |  |  |  |  |  |
| February | 0.103 | -0.013 |  |  |  |  |
|  | (0.237) | (0.117) |  |  |  |  |
| March | -0.605*** | -0.442** |  |  |  |  |
|  | (0.183) | (0.179) |  |  |  |  |
| April | -0.160 | 0.412 |  |  |  |  |
|  | (0.214) | (0.285) |  |  |  |  |
| November | 0.024 | 0.320** |  | -2.395*** | 0.456 |  |
|  | (0.155) | (0.131) |  | (0.222) | (0.334) |  |
| December | 0.175 | -0.411*** | 0.298*** | -2.277*** | -0.171 | -0.060 |
|  | (0.122) | (0.149) | (0.114) | (0.372) | (0.284) | (0.217) |
| Countries [*Ref: Mozambique*] | |  |  |  |  |  |
| Malawi | -0.200 |  |  |  |  |  |
|  | (0.222) |  |  |  |  |  |
| Rwanda | -0.270 |  |  |  |  |  |
|  | (0.182) |  |  |  |  |  |
| Uganda | -0.832** |  |  |  |  |  |
|  | (0.378) |  |  |  |  |  |
| Zambia | -0.003 |  |  |  |  |  |
|  | (0.178) |  |  |  |  |  |
| Observations | 19,541 | 2,591 | 3,973 | 4,744 | 3,996 | 4,237 |

Note: Standard errors in parentheses; *** p<0.01, ** p<0.05, * p<0.1; ^a^Ref categories; January (Pooled, Mozambique, Rwanda, Malawi, Uganda), November (Zambia)
