# Supplementary material for: What empowerment indicators are important for food consumption for women? Evidence from 5 sub-Sahara African countries
Source: PLoS One. 2021 Apr 21;16(4):e0250014. doi: 10.1371/journal.pone.0250014 (PMC8059862; doi:10.1371/journal.pone.0250014)
Supplement: S8 Table — (DOCX) [file pone.0250014.s008.docx]

S8 Table. Marginal effects of Poisson regression results for WDDS – Leadership domain (Group membership)

|  | (1) | (2) | (3) | (4) | (5) | (6) |
| --- | --- | --- | --- | --- | --- | --- |
| VARIABLES | All | Mozambique | Rwanda | Malawi | Uganda | Zambia |
| Membership of ≥ 1 group | 0.008 | 0.025 | 0.329** | 0.058 | -0.207* | 0.019 |
|  | (0.065) | (0.094) | (0.132) | (0.078) | (0.113) | (0.087) |
| SES index | -0.014 | 0.018 | 0.556 | -0.287** | -0.541 | -1.785** |
|  | (0.109) | (0.336) | (0.997) | (0.133) | (0.549) | (0.725) |
| SES index squared | 0.020 | 0.116 | 0.176 | 0.020 | 0.097 | -0.993** |
|  | (0.014) | (0.223) | (0.329) | (0.015) | (0.074) | (0.432) |
| Men’s age | 0.005*** | 0.007* | 0.002 | 0.005* | 0.008*** | 0.003 |
|  | (0.001) | (0.004) | (0.002) | (0.003) | (0.003) | (0.003) |
| Women’s age | -0.012*** | -0.011*** | -0.011*** | -0.016*** | -0.012*** | -0.003 |
|  | (0.002) | (0.004) | (0.004) | (0.003) | (0.004) | (0.003) |
| Women’s education | 0.042*** | 0.068 | 0.104*** | 0.084** | 0.032*** | 0.039*** |
|  | (0.010) | (0.060) | (0.030) | (0.037) | (0.011) | (0.013) |
| Household size | 0.031** | 0.050** | 0.050 | 0.036* | 0.016 | 0.043*** |
|  | (0.013) | (0.025) | (0.032) | (0.020) | (0.019) | (0.012) |
| Study location | -0.012** | 0.075*** | 0.021*** | 0.021 | -0.026*** | -0.075 |
|  | (0.005) | (0.016) | (0.008) | (0.056) | (0.007) | (0.073) |
| Study month^a^ |  |  |  |  |  |  |
| February | 0.108 | 0.172 |  |  |  |  |
|  | (0.251) | (0.124) |  |  |  |  |
| March | -0.576*** | -0.270 |  |  |  |  |
|  | (0.177) | (0.179) |  |  |  |  |
| April | -0.199 | 0.598* |  |  |  |  |
|  | (0.213) | (0.322) |  |  |  |  |
| November | 0.021 | 0.388*** |  | -2.380*** | 0.524 |  |
|  | (0.154) | (0.138) |  | (0.220) | (0.346) |  |
| December | 0.173 | -0.321** | 0.287** | -2.255*** | -0.058 | -0.047 |
|  | (0.120) | (0.157) | (0.116) | (0.369) | (0.288) | (0.219) |
| Countries [*Ref: Mozambique*] | |  |  |  |  |  |
| Malawi | -0.205 |  |  |  |  |  |
|  | (0.221) |  |  |  |  |  |
| Rwanda | -0.267 |  |  |  |  |  |
|  | (0.181) |  |  |  |  |  |
| Uganda | -0.868** |  |  |  |  |  |
|  | (0.379) |  |  |  |  |  |
| Zambia | 0.024 |  |  |  |  |  |
|  | (0.177) |  |  |  |  |  |
| Observations | 18,913 | 2,201 | 3,895 | 4,730 | 3,927 | 4,160 |

Note: Standard errors in parentheses; *** p<0.01, ** p<0.05, * p<0.1; ^a^Ref categories; January (Pooled, Mozambique, Rwanda, Malawi, Uganda), November (Zambia)
