# Supplementary material for: What empowerment indicators are important for food consumption for women? Evidence from 5 sub-Sahara African countries
Source: PLoS One. 2021 Apr 21;16(4):e0250014. doi: 10.1371/journal.pone.0250014 (PMC8059862; doi:10.1371/journal.pone.0250014)
Supplement: S9 Table — (DOCX) [file pone.0250014.s009.docx]

S9 Table. Marginal effects of Poisson for WDDS – Leadership domain (comfortable speaking in public in ≥1 context)

|  | (1) | (2) | (3) | (4) | (5) | (6) |
| --- | --- | --- | --- | --- | --- | --- |
| VARIABLES | All | Mozambique | Rwanda | Malawi | Uganda | Zambia |
| Public speaking | 0.149*** | 0.234** | 0.489*** | -0.080 | -0.057 | 0.098 |
|  | (0.057) | (0.121) | (0.111) | (0.075) | (0.128) | (0.089) |
| SES index | -0.009 | 0.020 | 0.802 | -0.284** | -0.653 | -1.771** |
|  | (0.107) | (0.316) | (0.961) | (0.129) | (0.546) | (0.698) |
| SES index squared | 0.017 | 0.129 | 0.245 | 0.020 | 0.112 | -0.990** |
|  | (0.014) | (0.213) | (0.318) | (0.015) | (0.073) | (0.419) |
| Men’s age | 0.006*** | 0.007* | 0.003 | 0.005* | 0.008*** | 0.003 |
|  | (0.001) | (0.004) | (0.002) | (0.003) | (0.003) | (0.003) |
| Women’s age | -0.011*** | -0.012*** | -0.010*** | -0.016*** | -0.012*** | -0.004 |
|  | (0.002) | (0.004) | (0.004) | (0.003) | (0.003) | (0.003) |
| Women’s education | 0.042*** | 0.009 | 0.118*** | 0.085** | 0.032*** | 0.040*** |
|  | (0.009) | (0.061) | (0.030) | (0.037) | (0.011) | (0.013) |
| Household size | 0.031** | 0.047* | 0.053* | 0.037* | 0.014 | 0.043*** |
|  | (0.013) | (0.026) | (0.030) | (0.020) | (0.019) | (0.012) |
| Study location | -0.014*** | 0.059*** | 0.019** | 0.019 | -0.027*** | -0.072 |
|  | (0.005) | (0.014) | (0.008) | (0.056) | (0.006) | (0.072) |
| Study month^a^ |  |  |  |  |  |  |
| February | 0.116 | -0.084 |  |  |  |  |
|  | (0.232) | (0.134) |  |  |  |  |
| March | -0.587*** | -0.511** |  |  |  |  |
|  | (0.181) | (0.204) |  |  |  |  |
| April | -0.150 | 0.319 |  |  |  |  |
|  | (0.213) | (0.303) |  |  |  |  |
| November | 0.034 | 0.229 |  | -2.382*** | 0.381 |  |
|  | (0.154) | (0.158) |  | (0.221) | (0.344) |  |
| December | 0.186 | -0.483*** | 0.330*** | -2.244*** | -0.165 | -0.052 |
|  | (0.121) | (0.160) | (0.109) | (0.372) | (0.279) | (0.217) |
| Countries [*Ref: Mozambique*] | |  |  |  |  |  |
| Malawi | -0.201 |  |  |  |  |  |
|  | (0.219) |  |  |  |  |  |
| Rwanda | -0.264 |  |  |  |  |  |
|  | (0.181) |  |  |  |  |  |
| Uganda | -0.858** |  |  |  |  |  |
|  | (0.375) |  |  |  |  |  |
| Zambia | -0.004 |  |  |  |  |  |
|  | (0.178) |  |  |  |  |  |
| Observations | 19,670 | 2,594 | 4,020 | 4,761 | 4,067 | 4,228 |

Note: Standard errors in parentheses; *** p<0.01, ** p<0.05, * p<0.1; ^a^Ref categories; January (Pooled, Mozambique, Rwanda, Malawi, Uganda), November (Zambia)
