# Supplementary material for: What empowerment indicators are important for food consumption for women? Evidence from 5 sub-Sahara African countries
Source: PLoS One. 2021 Apr 21;16(4):e0250014. doi: 10.1371/journal.pone.0250014 (PMC8059862; doi:10.1371/journal.pone.0250014)
Supplement: S10 Table — (DOCX) [file pone.0250014.s010.docx]

S10 Table. Marginal effects of Poisson for WDDS – Time domain (Non-excessive workload [<10.5hrs in 24hrs])

|  | (1) | (2) | (3) | (4) | (5) | (6) |
| --- | --- | --- | --- | --- | --- | --- |
| VARIABLES | All | Mozambique | Rwanda | Malawi | Uganda | Zambia |
| Non-excessive workload | -0.098* | -0.301** | -0.093 | -0.016 | -0.087 | -0.054 |
|  | (0.052) | (0.135) | (0.079) | (0.051) | (0.089) | (0.082) |
| SES index | -0.041 | -0.005 | 0.592 | -0.273** | -0.915** | -1.850** |
|  | (0.107) | (0.324) | (1.095) | (0.125) | (0.407) | (0.737) |
| SES index squared | 0.019 | 0.096 | 0.191 | 0.019 | 0.145*** | -1.039** |
|  | (0.015) | (0.210) | (0.359) | (0.015) | (0.056) | (0.439) |
| Men’s age | 0.005*** | 0.006* | 0.002 | 0.006* | 0.008*** | 0.003 |
|  | (0.002) | (0.004) | (0.002) | (0.003) | (0.003) | (0.003) |
| Women’s age | -0.012*** | -0.009** | -0.010*** | -0.015*** | -0.013*** | -0.003 |
|  | (0.002) | (0.004) | (0.004) | (0.003) | (0.004) | (0.003) |
| Women’s education | 0.041*** | 0.057 | 0.113*** | 0.081** | 0.030*** | 0.040*** |
|  | (0.009) | (0.061) | (0.033) | (0.037) | (0.010) | (0.013) |
| Household size | 0.041*** | 0.041 | 0.052 | 0.044** | 0.025 | 0.044*** |
|  | (0.012) | (0.025) | (0.034) | (0.022) | (0.017) | (0.012) |
| Study location | -0.014** | 0.073*** | 0.020** | 0.026 | -0.027*** | -0.079 |
|  | (0.006) | (0.016) | (0.008) | (0.057) | (0.007) | (0.072) |
| Study month^a^ |  |  |  |  |  |  |
| February | 0.043 | 0.054 |  |  |  |  |
|  | (0.251) | (0.136) |  |  |  |  |
| March | -0.592*** | -0.367* |  |  |  |  |
|  | (0.180) | (0.187) |  |  |  |  |
| April | -0.267 | 0.417 |  |  |  |  |
|  | (0.217) | (0.328) |  |  |  |  |
| November | -0.007 | 0.270* |  | -2.431*** | 0.391 |  |
|  | (0.159) | (0.151) |  | (0.230) | (0.315) |  |
| December | 0.152 | -0.398*** | 0.277** | -2.324*** | -0.169 | -0.047 |
|  | (0.126) | (0.145) | (0.118) | (0.379) | (0.203) | (0.213) |
| Countries [*Ref: Mozambique*] | |  |  |  |  |  |
| Malawi | -0.258 |  |  |  |  |  |
|  | (0.219) |  |  |  |  |  |
| Rwanda | -0.309* |  |  |  |  |  |
|  | (0.183) |  |  |  |  |  |
| Uganda | -0.784** |  |  |  |  |  |
|  | (0.373) |  |  |  |  |  |
| Zambia | -0.074 |  |  |  |  |  |
|  | (0.178) |  |  |  |  |  |
| Observations | 18,117 | 2,100 | 3,681 | 4,569 | 3,754 | 4,013 |

Note: Standard errors in parentheses; *** p<0.01, ** p<0.05, * p<0.1; ^a^Ref categories; January (Pooled, Mozambique, Rwanda, Malawi, Uganda), November (Zambia)
