# Supplementary material for: What empowerment indicators are important for food consumption for women? Evidence from 5 sub-Sahara African countries
Source: PLoS One. 2021 Apr 21;16(4):e0250014. doi: 10.1371/journal.pone.0250014 (PMC8059862; doi:10.1371/journal.pone.0250014)
Supplement: S11 Table — (DOCX) [file pone.0250014.s011.docx]

S11 Table. Marginal effects of Poisson regression for WDDS – Time domain (satisfaction with leisure time)

|  | (1) | (2) | (3) | (4) | (5) | (6) |
| --- | --- | --- | --- | --- | --- | --- |
| VARIABLES | All | Mozambique | Rwanda | Malawi | Uganda | Zambia |
| Stfsd with leisure time | -0.008 | -0.068 | -0.012 | 0.148* | -0.037 | -0.015 |
|  | (0.075) | (0.149) | (0.102) | (0.086) | (0.102) | (0.093) |
| SES index | -0.014 | -0.014 | 0.639 | -0.293** | -0.676 | -1.761** |
|  | (0.108) | (0.326) | (1.037) | (0.130) | (0.540) | (0.698) |
| SES index squared | 0.018 | 0.110 | 0.194 | 0.021 | 0.115 | -0.981** |
|  | (0.014) | (0.220) | (0.341) | (0.015) | (0.072) | (0.418) |
| Men’s age | 0.005*** | 0.007* | 0.003 | 0.006* | 0.008*** | 0.003 |
|  | (0.001) | (0.004) | (0.002) | (0.003) | (0.003) | (0.003) |
| Women’s age | -0.011*** | -0.012*** | -0.010*** | -0.016*** | -0.012*** | -0.003 |
|  | (0.002) | (0.004) | (0.004) | (0.003) | (0.003) | (0.003) |
| Women’s education | 0.042*** | 0.011 | 0.120*** | 0.083** | 0.032*** | 0.040*** |
|  | (0.009) | (0.061) | (0.030) | (0.037) | (0.010) | (0.013) |
| Household size | 0.032** | 0.048* | 0.048 | 0.036* | 0.013 | 0.044*** |
|  | (0.013) | (0.025) | (0.032) | (0.020) | (0.019) | (0.012) |
| Study location | -0.014*** | 0.060*** | 0.018** | 0.019 | -0.027*** | -0.075 |
|  | (0.005) | (0.013) | (0.008) | (0.056) | (0.006) | (0.070) |
| Study month^a^ |  |  |  |  |  |  |
| February | 0.097 | -0.009 |  |  |  |  |
|  | (0.234) | (0.116) |  |  |  |  |
| March | -0.611*** | -0.444** |  |  |  |  |
|  | (0.182) | (0.179) |  |  |  |  |
| April | -0.163 | 0.412 |  |  |  |  |
|  | (0.214) | (0.285) |  |  |  |  |
| November | 0.022 | 0.324** |  | -2.355*** | 0.398 |  |
|  | (0.155) | (0.130) |  | (0.225) | (0.351) |  |
| December | 0.173 | -0.409*** | 0.293*** | -2.233*** | -0.150 | -0.059 |
|  | (0.121) | (0.151) | (0.113) | (0.373) | (0.284) | (0.212) |
| Countries [*Ref: Mozambique*] | |  |  |  |  |  |
| Malawi | -0.205 |  |  |  |  |  |
|  | (0.222) |  |  |  |  |  |
| Rwanda | -0.274 |  |  |  |  |  |
|  | (0.183) |  |  |  |  |  |
| Uganda | -0.838** |  |  |  |  |  |
|  | (0.379) |  |  |  |  |  |
| Zambia | -0.006 |  |  |  |  |  |
|  | (0.180) |  |  |  |  |  |
| Observations | 19,652 | 2,587 | 4,004 | 4,763 | 4,063 | 4,235 |

Note: Standard errors in parentheses; *** p<0.01, ** p<0.05, * p<0.1; ^a^Ref categories; January (Pooled, Mozambique, Rwanda, Malawi, Uganda), November (Zambia)
