# Supplementary material for: What empowerment indicators are important for food consumption for women? Evidence from 5 sub-Sahara African countries
Source: PLoS One. 2021 Apr 21;16(4):e0250014. doi: 10.1371/journal.pone.0250014 (PMC8059862; doi:10.1371/journal.pone.0250014)
Supplement: S12 Table — (DOCX) [file pone.0250014.s012.docx]

S12 Table. Marginal effects of Logistic regression for food groups consumed – Production domain (Autonomy in ≥ 1 activity linked to production) – Pooled

|  | (1) | (2) | (3) | (4) | (5) | (6) | (7) | (8) | (9) |
| --- | --- | --- | --- | --- | --- | --- | --- | --- | --- |
|  | Grains/  Roots | Legumes | Dairy | Organ meat | Eggs | Flesh protein | Dark-green leafy veg | Other vit A-rich frts/vegs | Other frts/vegs |
| Aut in prod decs | -0.013* | -0.025** | 0.008 | 0.003 | 0.024*** | 0.023* | 0.056*** | 0.088*** | 0.018 |
|  | (0.007) | (0.011) | (0.009) | (0.004) | (0.008) | (0.012) | (0.013) | (0.013) | (0.013) |
| SES index | -0.003 | -0.029** | 0.061*** | -0.013** | 0.004 | 0.002 | 0.017 | 0.024** | 0.027** |
|  | (0.006) | (0.01) | (0.009) | (0.004) | (0.005) | (0.011) | (0.012) | (0.012) | (0.012) |
| SES index squared | 0.002* | 0.002 | 0.006** | 0.002*** | 0.001 | 0.006*** | 0.005** | 0.004** | 0.013*** |
|  | (0.001) | (0.002) | (0.001) | (0.001) | (0.001) | (0.002) | (0.002) | (0.002) | (0.002) |
| Men’s age | 0.000 | 0.001*** | 0.001*** | 0.000 | 0.000** | 0.001** | 0.000 | 0.001 | 0.001*** |
|  | (0.000) | (0.000) | (0.000) | (0.000) | (0.000) | (0.000) | (0.000) | (0.000) | (0.000) |
| Women’s age | 0.000* | 0.000 | -0.001** | 0.000** | -0.002** | -0.001** | 0.000 | -0.005** | -0.002*** |
|  | (0.000) | (0.000) | (0.000) | (0.000) | (0.000) | (0.000) | (0.000) | (0.000) | (0.000) |
| Women’s education | 0.001 | 0.003 | 0.011*** | 0.001** | 0.004*** | 0.007*** | -0.002 | 0.006*** | 0.011*** |
|  | (0.001) | (0.002) | (0.001) | (0.000) | (0.001) | (0.002) | (0.002) | (0.002) | (0.002) |
| Household size | 0.002*** | -0.002 | 0.004*** | -0.001** | 0.001 | 0.003** | 0.015*** | 0.007*** | 0.003** |
|  | (0.001) | (0.001) | (0.001) | (0.000) | (0.001) | (0.002) | (0.002) | (0.002) | (0.002) |
| Study location | 0.000 | -0.001** | -0.003** | 0.000*** | -0.002** | -0.003** | -0.004** | 0.001** | -0.001** |
|  | (0.000) | (0.000) | (0.000) | (0.000) | (0.000) | (0.001) | (0.001) | (0.001) | (0.001) |
| Study month [*Ref: January*] | |  |  |  |  |  |  |  |  |
| February | -0.072*** | -0.112** | -0.004 | 0.014** | 0.079*** | 0.172*** | 0.058* | 0.126*** | -0.208*** |
|  | (0.012) | (0.034) | (0.011) | (0.006) | (0.018) | (0.031) | (0.032) | (0.036) | (0.031) |
| March | -0.084*** | -0.193** | -0.017 | 0.022** | -0.025 | 0.167*** | -0.094** | -0.07* | -0.395*** |
|  | (0.018) | (0.039) | (0.013) | (0.011) | (0.018) | (0.039) | (0.039) | (0.04) | (0.037) |
| April | -0.045*** | -0.122** | -0.011 | 0.007 | 0.036 | 0.133*** | -0.056 | 0.044 | -0.175*** |
|  | (0.016) | (0.044) | (0.017) | (0.007) | (0.028) | (0.043) | (0.043) | (0.045) | (0.043) |
| November | -0.001 | -0.027 | 0.034*** | 0.005 | -0.013 | -0.221** | 0.12*** | 0.028 | 0.066*** |
|  | (0.009) | (0.017) | (0.012) | (0.007) | (0.008) | (0.018) | (0.02) | (0.02) | (0.018) |
| December | 0.007 | -0.02* | 0.013 | 0.002 | 0.01** | -0.048** | -0.022 | 0.154*** | 0.078*** |
|  | (0.008) | (0.011) | (0.011) | (0.005) | (0.005) | (0.011) | (0.016) | (0.016) | (0.014) |
| Countries [*Ref: Mozambique*] | |  |  |  |  |  |  |  |  |
| Malawi | -0.085*** | 0.42*** | 0.187*** | 0.01 | -0.05*** | -0.217** | -0.106** | 0.234*** | -0.607*** |
|  | (0.01) | (0.033) | (0.014) | (0.006) | (0.016) | (0.03) | (0.032) | (0.035) | (0.029) |
| Rwanda | -0.027*** | -0.064** | -0.016** | 0.014*** | -0.008 | 0.202*** | -0.101** | 0.334*** | -0.619*** |
|  | (0.004) | (0.031) | (0.007) | (0.004) | (0.015) | (0.028) | (0.027) | (0.031) | (0.025) |
| Uganda | -0.08*** | 0.485*** | -0.123** | 0.035*** | -0.071** | -0.137** | -0.266** | -0.032 | -0.658*** |
|  | (0.021) | (0.044) | (0.028) | (0.012) | (0.022) | (0.046) | (0.049) | (0.05) | (0.045) |
| Zambia | -0.019*** | 0.033 | -0.013 | 0.035*** | 0.024 | 0.239*** | -0.244** | 0.511*** | -0.538*** |
|  | (0.005) | (0.032) | (0.01) | (0.005) | (0.017) | (0.029) | (0.03) | (0.032) | (0.027) |
| Observations | 19756 | 19756 | 19756 | 19756 | 19756 | 19756 | 19756 | 19756 | 19756 |
| Standard errors in parentheses; *** p<0.01, ** p<0.05, * p<0.1 | | | | | | | | | |
|  | | | | | | | | | |
|  | | | | | | | | | |
