# Supplementary material for: What empowerment indicators are important for food consumption for women? Evidence from 5 sub-Sahara African countries
Source: PLoS One. 2021 Apr 21;16(4):e0250014. doi: 10.1371/journal.pone.0250014 (PMC8059862; doi:10.1371/journal.pone.0250014)
Supplement: S13 Table — (DOCX) [file pone.0250014.s013.docx]

S13 Table. Marginal effects of Logistic regression for food groups consumed – Production domain (Input in ≥ 2 productive decisions) – Pooled

|  | (1) | (2) | (3) | (4) | (5) | (6) | (7) | (8) | (9) |
| --- | --- | --- | --- | --- | --- | --- | --- | --- | --- |
| VARIABLES | Grains | Legumes | Dairy | Organ meat | Eggs | Flesh protein | Vit A-rich leafy green | Othr vit A-rich fruit/veg | Other fruit/veg |
| Input prod dec | 0.015** | 0.031* | 0.039** | 0.002 | 0.017* | 0.037* | 0.021** | 0.049** | 0.053** |
|  | (0.008) | (0.017) | (0.018) | (0.004) | (0.009) | (0.019) | (0.022) | (0.021) | (0.022) |
| SES index | -0.012 | 0.016 | 0.010 | -0.009*** | -0.006 | -0.024* | 0.003 | -0.146*** | -0.036** |
|  | (0.008) | (0.012) | (0.012) | (0.003) | (0.006) | (0.014) | (0.014) | (0.014) | (0.016) |
| SES index sqd | 0.003 | 0.001 | -0.000 | 0.001*** | 0.002 | 0.007* | -0.004 | 0.017*** | 0.012*** |
|  | (0.003) | (0.004) | (0.003) | (0.001) | (0.002) | (0.004) | (0.004) | (0.004) | (0.004) |
| Men’s age | 0.000 | 0.002*** | 0.001*** | 0.000 | 0.000 | 0.000 | -0.000 | 0.000 | 0.001** |
|  | (0.000) | (0.001) | (0.000) | (0.000) | (0.000) | (0.001) | (0.001) | (0.001) | (0.001) |
| Women’s age | -0.000 | 0.000 | -0.001 | 0.000 | -0.002*** | -0.002** | -0.000 | -0.005*** | -0.003*** |
|  | (0.000) | (0.001) | (0.001) | (0.000) | (0.000) | (0.001) | (0.001) | (0.001) | (0.001) |
| Women’s educ | -0.000 | 0.009*** | 0.012*** | 0.001 | 0.003 | 0.001 | -0.002 | 0.001 | 0.011*** |
|  | (0.002) | (0.003) | (0.003) | (0.001) | (0.002) | (0.003) | (0.004) | (0.003) | (0.004) |
| Household size | 0.002 | 0.000 | 0.003 | -0.001 | 0.001 | 0.002 | 0.014** | 0.006 | 0.003 |
|  | (0.001) | (0.004) | (0.003) | (0.001) | (0.002) | (0.004) | (0.006) | (0.005) | (0.004) |
| Study location | -0.001 | 0.001 | -0.003** | -0.000 | -0.004*** | -0.005*** | -0.004** | 0.001 | -0.001 |
|  | (0.001) | (0.001) | (0.001) | (0.000) | (0.001) | (0.002) | (0.002) | (0.002) | (0.002) |
| Study month [*Ref: January*] | |  |  |  |  |  |  |  |  |
| February | 0.041** | -0.642*** | -0.148*** | 0.002 | 0.285*** | 0.551*** | 0.159** | 0.194*** | 0.411*** |
|  | (0.020) | (0.041) | (0.033) | (0.012) | (0.078) | (0.057) | (0.062) | (0.060) | (0.073) |
| March | 0.036 | -0.698*** | -0.150*** | 0.018 | 0.088* | 0.548*** | -0.035 | -0.019 | 0.236*** |
|  | (0.025) | (0.040) | (0.033) | (0.027) | (0.049) | (0.076) | (0.083) | (0.078) | (0.072) |
| April | 0.053*** | -0.626*** | -0.143*** | -0.007 | 0.148** | 0.503*** | -0.003 | 0.086 | 0.441*** |
|  | (0.018) | (0.057) | (0.034) | (0.012) | (0.061) | (0.085) | (0.102) | (0.098) | (0.077) |
| November | 0.042** | -0.393*** | -0.088** | -0.001 | 0.022 | 0.069 | 0.190*** | 0.067 | 0.086 |
|  | (0.019) | (0.045) | (0.037) | (0.007) | (0.018) | (0.047) | (0.044) | (0.060) | (0.053) |
| December | 0.016 | -0.112*** | -0.030 | -0.001 | 0.021 | 0.019 | -0.015 | 0.122*** | 0.091*** |
|  | (0.018) | (0.029) | (0.037) | (0.007) | (0.016) | (0.048) | (0.049) | (0.046) | (0.035) |
| Countries [*Ref: Mozambique*] | |  |  |  |  |  |  |  |  |
| Malawi | -0.088*** | 0.418*** | 0.185*** | 0.011 | -0.054*** | -0.213*** | -0.102*** | 0.228*** | -0.616*** |
|  | (0.01) | (0.033) | (0.014) | (0.007) | (0.016) | (0.03) | (0.033) | (0.035) | (0.03) |
| Rwanda | -0.036*** | -0.066** | -0.025*** | 0.015*** | -0.017 | 0.198*** | -0.084*** | 0.339*** | -0.63*** |
|  | (0.004) | (0.031) | (0.007) | (0.004) | (0.015) | (0.028) | (0.028) | (0.031) | (0.025) |
| Uganda | -0.089*** | 0.475*** | -0.125*** | 0.031** | -0.076*** | -0.153*** | -0.236*** | -0.026 | -0.668*** |
|  | (0.022) | (0.045) | (0.028) | (0.012) | (0.022) | (0.047) | (0.05) | (0.051) | (0.046) |
| Zambia | -0.026*** | 0.027 | -0.023** | 0.036*** | 0.017 | 0.236*** | -0.231*** | 0.511*** | -0.553*** |
|  | (0.006) | (0.033) | (0.01) | (0.005) | (0.017) | (0.03) | (0.031) | (0.032) | (0.027) |
| Observations | 19,303 | 19,303 | 19,303 | 19,303 | 19,303 | 19,303 | 19,303 | 19,303 | 19,303 |

Standard errors in parentheses; *** p<0.01, ** p<0.05, * p<0.1
