# Supplementary material for: What empowerment indicators are important for food consumption for women? Evidence from 5 sub-Sahara African countries
Source: PLoS One. 2021 Apr 21;16(4):e0250014. doi: 10.1371/journal.pone.0250014 (PMC8059862; doi:10.1371/journal.pone.0250014)
Supplement: S14 Table — (DOCX) [file pone.0250014.s014.docx]

S14 Table. Marginal effects of Logistic regression for food groups consumed – Production domain (Input in ≥ 2 productive decisions) - Rwanda

|  | (1) | (2) | (3) | (4) | (5) | (6) | (7) | (8) | (9) |
| --- | --- | --- | --- | --- | --- | --- | --- | --- | --- |
| VARIABLES | Grains | Legumes | Dairy | Organ meat | Eggs | Flesh protein | Vit A-rich leafy green | Othr vit A-rich fruit/veg | Other fruit/veg |
| Input in prod decs | 0.041*** | 0.044* | 0.085*** | 0.006 | 0.003 | 0.087*** | 0.053 | 0.113*** | 0.114*** |
|  | (0.014) | (0.027) | (0.025) | (0.009) | (0.011) | (0.026) | (0.038) | (0.034) | (0.029) |
| SES index | 0.066 | -0.197 | 0.141 | 0.130** | 0.009 | 0.256 | 0.013 | -0.303 | 0.430 |
|  | (0.182) | (0.254) | (0.233) | (0.065) | (0.066) | (0.277) | (0.344) | (0.426) | (0.323) |
| SES index squared | 0.026 | -0.045 | -0.003 | 0.048** | 0.001 | 0.069 | 0.003 | -0.087 | 0.153 |
|  | (0.063) | (0.089) | (0.086) | (0.023) | (0.024) | (0.094) | (0.117) | (0.146) | (0.112) |
| Men’s age | -0.000 | -0.000 | 0.000 | -0.000 | 0.000 | 0.000 | 0.001 | 0.001 | -0.000 |
|  | (0.000) | (0.001) | (0.001) | (0.000) | (0.000) | (0.000) | (0.001) | (0.001) | (0.001) |
| Women’s age | -0.000 | -0.000 | -0.001 | 0.001 | -0.002** | 0.001 | -0.003** | -0.004*** | -0.002 |
|  | (0.001) | (0.001) | (0.001) | (0.000) | (0.001) | (0.001) | (0.001) | (0.001) | (0.001) |
| Women’s education | 0.012 | 0.004 | 0.017** | 0.003 | 0.001 | 0.001 | 0.031* | 0.024* | 0.023** |
|  | (0.011) | (0.009) | (0.007) | (0.002) | (0.002) | (0.008) | (0.016) | (0.013) | (0.010) |
| Household size | -0.001 | 0.004 | 0.016** | -0.001 | 0.002 | 0.002 | 0.001 | 0.004 | 0.005 |
|  | (0.005) | (0.006) | (0.007) | (0.002) | (0.003) | (0.006) | (0.013) | (0.011) | (0.008) |
| Study location | 0.001 | 0.002 | -0.003 | 0.000 | 0.000 | 0.003 | 0.007** | 0.005* | 0.006** |
|  | (0.001) | (0.002) | (0.002) | (0.001) | (0.001) | (0.002) | (0.003) | (0.003) | (0.003) |
| Study month [*Ref: January*] | | |  |  |  |  |  |  |  |
| December | 0.013 | -0.015 | 0.018 | 0.001 | 0.016 | -0.037 | -0.016 | 0.166*** | 0.085** |
|  | (0.020) | (0.027) | (0.034) | (0.010) | (0.011) | (0.031) | (0.046) | (0.052) | (0.038) |
| Observations | 3,848 | 3,848 | 3,848 | 3,848 | 3,848 | 3,848 | 3,848 | 3,848 | 3,848 |

Standard errors in parentheses; *** p<0.01, ** p<0.05, * p<0.1
