# Supplementary material for: What empowerment indicators are important for food consumption for women? Evidence from 5 sub-Sahara African countries
Source: PLoS One. 2021 Apr 21;16(4):e0250014. doi: 10.1371/journal.pone.0250014 (PMC8059862; doi:10.1371/journal.pone.0250014)
Supplement: S15 Table — (DOCX) [file pone.0250014.s015.docx]

S15 Table. Marginal effects of Logistic regression for food groups consumed – Leadership domain (Comfortable speaking in public in ≥ 1 context) – Pooled

|  | (1) | (2) | (3) | (4) | (5) | (6) | (7) | (8) | (9) |
| --- | --- | --- | --- | --- | --- | --- | --- | --- | --- |
| VARIABLES | Grains | Legumes | Dairy | Organ meat | Eggs | Flesh protein | Vit A-rich leafy green | Othr vit A-rich fruit/veg | Other fruit/veg |
| Public speaking | 0.018* | -0.004 | -0.005 | 0.004 | -0.003 | 0.044 | 0.012 | 0.021** | 0.072*** |
|  | (0.010) | (0.016) | (0.021) | (0.004) | (0.011) | (0.021) | (0.027) | (0.023) | (0.020) |
| SES index | -0.012 | 0.016 | 0.008 | 0.009*** | -0.007 | 0.026* | 0.005 | 0.145*** | 0.038** |
|  | (0.007) | (0.012) | (0.012) | (0.003) | (0.006) | (0.013) | (0.014) | (0.014) | (0.015) |
| SES index sqd | 0.002 | 0.001 | 0.000 | 0.001*** | 0.002 | 0.006 | -0.004 | 0.017*** | 0.013*** |
|  | (0.002) | (0.004) | (0.003) | (0.001) | (0.002) | (0.004) | (0.004) | (0.004) | (0.004) |
| Men’s age | 0.000 | 0.002*** | 0.001*** | 0.000 | 0.000* | 0.000 | -0.000 | 0.000 | 0.001** |
|  | (0.000) | (0.001) | (0.000) | (0.000) | (0.000) | (0.001) | (0.001) | (0.001) | (0.001) |
| Women’s age | -0.000 | 0.000 | -0.001 | 0.000 | -0.002*** | -0.002** | -0.000 | -0.005*** | -0.002*** |
|  | (0.000) | (0.001) | (0.001) | (0.000) | (0.000) | (0.001) | (0.001) | (0.001) | (0.001) |
| Women’s educ | 0.000 | 0.009*** | 0.012*** | 0.001 | 0.004** | 0.001 | -0.003 | 0.002 | 0.011*** |
|  | (0.002) | (0.003) | (0.003) | (0.001) | (0.002) | (0.003) | (0.004) | (0.003) | (0.004) |
| Household size | 0.002 | -0.000 | 0.003 | -0.001 | 0.001 | 0.002 | 0.015** | 0.006 | 0.003 |
|  | (0.001) | (0.004) | (0.003) | (0.001) | (0.002) | (0.004) | (0.006) | (0.005) | (0.004) |
| Study location | -0.001 | 0.001 | -0.002** | -0.000* | -0.003** | -0.005*** | -0.004** | 0.000 | -0.001 |
|  | (0.001) | (0.001) | (0.001) | (0.000) | (0.001) | (0.002) | (0.002) | (0.001) | (0.002) |
| Study month [*Ref: January*] | |  |  |  |  |  |  |  |  |
| February | 0.042** | -0.638*** | -0.143*** | 0.001 | 0.271*** | 0.551*** | 0.159** | 0.180*** | 0.416*** |
|  | (0.021) | (0.039) | (0.032) | (0.012) | (0.073) | (0.058) | (0.062) | (0.061) | (0.072) |
| March | 0.037 | -0.696*** | -0.144*** | 0.016 | 0.083* | 0.544*** | -0.021 | -0.033 | 0.235*** |
|  | (0.025) | (0.038) | (0.032) | (0.026) | (0.047) | (0.078) | (0.082) | (0.077) | (0.071) |
| April | 0.055*** | -0.623*** | -0.138*** | -0.007 | 0.161** | 0.503*** | 0.013 | 0.073 | 0.441*** |
|  | (0.018) | (0.053) | (0.033) | (0.011) | (0.071) | (0.084) | (0.100) | (0.098) | (0.077) |
| November | 0.046** | -0.384*** | -0.077** | 0.001 | 0.035* | 0.078* | 0.195*** | 0.076 | 0.104** |
|  | (0.019) | (0.045) | (0.036) | (0.007) | (0.019) | (0.047) | (0.043) | (0.058) | (0.053) |
| December | 0.018 | -0.098*** | -0.022 | -0.001 | 0.028** | 0.021 | -0.012 | 0.127*** | 0.103*** |
|  | (0.019) | (0.031) | (0.036) | (0.007) | (0.014) | (0.048) | (0.048) | (0.045) | (0.033) |
| Countries [*Ref: Mozambique*] | |  |  |  |  |  |  |  |  |
| Malawi | -0.083*** | 0.42*** | 0.188*** | 0.01 | -0.049*** | -0.215*** | -0.104*** | 0.235*** | -0.604*** |
|  | (0.01) | (0.033) | (0.014) | (0.006) | (0.016) | (0.03) | (0.032) | (0.035) | (0.029) |
| Rwanda | -0.028*** | -0.066** | -0.014** | 0.015*** | -0.005 | 0.209*** | -0.093*** | 0.349*** | -0.612*** |
|  | (0.004) | (0.031) | (0.007) | (0.004) | (0.015) | (0.028) | (0.027) | (0.031) | (0.025) |
| Uganda | -0.087*** | 0.482*** | -0.123*** | 0.032*** | -0.071*** | -0.147*** | -0.269*** | -0.033 | -0.665*** |
|  | (0.021) | (0.044) | (0.028) | (0.012) | (0.022) | (0.046) | (0.049) | (0.05) | (0.045) |
| Zambia | -0.02*** | 0.031 | -0.012 | 0.036*** | 0.025 | 0.242*** | -0.242*** | 0.517*** | -0.536*** |
|  | (0.005) | (0.032) | (0.01) | (0.005) | (0.017) | (0.029) | (0.03) | (0.032) | (0.027) |
| Observations | 19,758 | 19,758 | 19,758 | 19,758 | 19,758 | 19,758 | 19,758 | 19,758 | 19,758 |

Standard errors in parentheses; *** p<0.01, ** p<0.05, * p<0.1
