# Supplementary material for: What empowerment indicators are important for food consumption for women? Evidence from 5 sub-Sahara African countries
Source: PLoS One. 2021 Apr 21;16(4):e0250014. doi: 10.1371/journal.pone.0250014 (PMC8059862; doi:10.1371/journal.pone.0250014)
Supplement: S16 Table — (DOCX) [file pone.0250014.s016.docx]

S16 Table. Marginal effects of Logistic regression for food groups consumed – Leadership domain (Comfortable speaking in public in ≥ 1 context) - Mozambique

|  | (1) | (2) | (3) | (4) | (5) | (6) | (7) | (8) | (9) |
| --- | --- | --- | --- | --- | --- | --- | --- | --- | --- |
| VARIABLES | Grains | Legumes | Dairy | Organ meat | Eggs | Flesh protein | Vit A-rich leafy green | Othr vit A-rich fruit/veg | Other fruit/veg |
| Public speaking | 0.014 | 0.017 | -0.005 | 0.003 | 0.035 | 0.063 | 0.049 | 0.119*** | 0.075** |
|  | (0.012) | (0.035) | (0.005) | (0.006) | (0.025) | (0.050) | (0.034) | (0.040) | (0.037) |
| SES index | -0.022 | -0.039 | 0.001 | 0.009 | 0.038 | 0.034 | 0.099 | 0.044** | 0.032*** |
|  | (0.026) | (0.069) | (0.008) | (0.006) | (0.029) | (0.067) | (0.061) | (0.070) | (0.068) |
| SES index squrd | -0.002 | -0.121* | 0.007 | 0.007 | 0.055* | 0.070 | 0.163*** | 0.018*** | 0.057*** |
|  | (0.022) | (0.066) | (0.005) | (0.005) | (0.033) | (0.056) | (0.055) | (0.071) | (0.062) |
| Men’s age | 0.002** | 0.002 | 0.000 | -0.000 | 0.000 | 0.001 | -0.001 | 0.000 | 0.001 |
|  | (0.001) | (0.002) | (0.000) | (0.000) | (0.001) | (0.001) | (0.001) | (0.001) | (0.002) |
| Women’s age | -0.001 | -0.000 | -0.000 | -0.000* | -0.001 | -0.002 | 0.000 | -0.004** | -0.003* |
|  | (0.001) | (0.002) | (0.000) | (0.000) | (0.001) | (0.002) | (0.002) | (0.002) | (0.002) |
| Women’s education | 0.035*** | 0.009 | -0.001 | -0.002 | 0.011 | 0.002 | -0.007 | -0.009 | -0.005 |
|  | (0.014) | (0.018) | (0.004) | (0.003) | (0.009) | (0.017) | (0.020) | (0.015) | (0.018) |
| Household size | 0.005 | 0.017* | 0.001 | 0.000 | 0.002 | -0.001 | 0.010 | 0.008 | 0.017* |
|  | (0.004) | (0.009) | (0.001) | (0.001) | (0.005) | (0.010) | (0.008) | (0.008) | (0.009) |
| Study location | 0.009*** | 0.002 | -0.000 | 0.000 | 0.005** | 0.004 | 0.002 | 0.017*** | 0.014*** |
|  | (0.002) | (0.003) | (0.001) | (0.001) | (0.002) | (0.005) | (0.003) | (0.004) | (0.004) |
| Study month [*Ref: November*] | |  |  |  |  |  |  |  |  |
| December | 0.039** | 0.010* | -0.000 | -0.002* | -0.009* | -0.033*** | -0.009* | -0.003 | 0.027*** |
|  | (0.018) | (0.006) | (0.001) | (0.001) | (0.005) | (0.005) | (0.005) | (0.007) | (0.008) |
| Observations | 2,604 | 2,604 | 2,604 | 2,604 | 2,604 | 2,604 | 2,604 | 2,604 | 2,604 |

Standard errors in parentheses; *** p<0.01, ** p<0.05, * p<0.1
