# Supplementary material for: What empowerment indicators are important for food consumption for women? Evidence from 5 sub-Sahara African countries
Source: PLoS One. 2021 Apr 21;16(4):e0250014. doi: 10.1371/journal.pone.0250014 (PMC8059862; doi:10.1371/journal.pone.0250014)
Supplement: S17 Table — (DOCX) [file pone.0250014.s017.docx]

S17 Table. Marginal effects of Logistic regression for food groups consumed – Leadership domain (Comfortable speaking in public in ≥ 1 context) - Rwanda

|  | (1) | (2) | (3) | (4) | (5) | (6) | (7) | (8) | (9) |
| --- | --- | --- | --- | --- | --- | --- | --- | --- | --- |
| VARIABLES | Grains | Legumes | Dairy | Organ meat | Eggs | Flesh protein | Vit A-rich leafy green | Othr vit A-rich fruit/veg | Other fruit/veg |
| Public speaking | 0.049** | 0.021 | 0.008 | 0.013 | -0.003 | 0.124 | 0.157*** | 0.066 | 0.091** |
|  | (0.020) | (0.025) | (0.040) | (0.009) | (0.008) | (0.039) | (0.039) | (0.047) | (0.040) |
| SES index | 0.047** | -0.211 | 0.086 | 0.125** | 0.003 | 0.286** | 0.091 | 0.287 | 0.384** |
|  | (0.171) | (0.253) | (0.230) | (0.062) | (0.060) | (0.254) | (0.334) | (0.419) | (0.307) |
| SES index squrd | 0.019** | -0.050 | -0.019 | 0.047** | -0.001 | 0.083*** | 0.025 | 0.082 | 0.137** |
|  | (0.059) | (0.089) | (0.083) | (0.022) | (0.022) | (0.086) | (0.115) | (0.144) | (0.106) |
| Men’s age | -0.000 | -0.000 | 0.000 | -0.000 | 0.000 | 0.000 | 0.002* | 0.001 | 0.000 |
|  | (0.000) | (0.001) | (0.001) | (0.000) | (0.000) | (0.000) | (0.001) | (0.001) | (0.001) |
| Women’s age | -0.000 | -0.000 | -0.001 | 0.001* | -0.002** | 0.001 | -0.003** | -0.005*** | -0.002 |
|  | (0.001) | (0.001) | (0.001) | (0.000) | (0.001) | (0.001) | (0.001) | (0.001) | (0.001) |
| Women’s education | 0.013 | 0.004 | 0.018*** | 0.003 | 0.001 | 0.003 | 0.032** | 0.030** | 0.023** |
|  | (0.011) | (0.010) | (0.007) | (0.002) | (0.002) | (0.007) | (0.015) | (0.014) | (0.009) |
| Household size | 0.002 | 0.005 | 0.017** | -0.000 | 0.002 | 0.006 | 0.005 | 0.009 | 0.008 |
|  | (0.005) | (0.006) | (0.008) | (0.002) | (0.002) | (0.007) | (0.012) | (0.011) | (0.008) |
| Study location | 0.001 | 0.001 | -0.003 | 0.000 | 0.000 | 0.003 | 0.007** | 0.005 | 0.006** |
|  | (0.001) | (0.002) | (0.002) | (0.001) | (0.001) | (0.002) | (0.003) | (0.003) | (0.003) |
| Study month [*Ref: January*] | | |  |  |  |  |  |  |  |
| December | 0.002 | -0.000 | 0.002 | 0.000 | 0.001* | -0.003 | 0.000 | 0.016*** | 0.009*** |
|  | (0.002) | (0.003) | (0.003) | (0.001) | (0.001) | (0.003) | (0.004) | (0.005) | (0.003) |
| Observations | 4,036 | 4,036 | 4,036 | 4,036 | 4,036 | 4,036 | 4,036 | 4,036 | 4,036 |

Standard errors in parentheses; *** p<0.01, ** p<0.05, * p<0.1
