# Supplementary material for: What empowerment indicators are important for food consumption for women? Evidence from 5 sub-Sahara African countries
Source: PLoS One. 2021 Apr 21;16(4):e0250014. doi: 10.1371/journal.pone.0250014 (PMC8059862; doi:10.1371/journal.pone.0250014)
Supplement: S18 Table — (DOCX) [file pone.0250014.s018.docx]

S18 Table. Correlation matrix – Pooled data

|  | SES index | Roof type | Floor type | Ext wall type | Toilet type | Source drinking water |
| --- | --- | --- | --- | --- | --- | --- |
| SES index | 1.0000 |  |  |  |  |  |
| Roof type | 0.7882 | 1.0000 |  |  |  |  |
| Floor type | 0.9387 | 0.6412 | 1.0000 |  |  |  |
| Ex wall type | 0.6654 | 0.4245 | 0.5924 | 1.0000 |  |  |
| Toilet type | 0.8714 | 0.6044 | 0.8009 | 0.4145 | 1.0000 |  |
| Source drinking water | 0.9073 | 0.6431 | 0.8534 | 0.4716 | 0.7718 | 1.0000 |
