# Supplementary material for: What empowerment indicators are important for food consumption for women? Evidence from 5 sub-Sahara African countries
Source: PLoS One. 2021 Apr 21;16(4):e0250014. doi: 10.1371/journal.pone.0250014 (PMC8059862; doi:10.1371/journal.pone.0250014)
Supplement: Attachment — Submitted filename: Response letter to reviewers PLOS 2.docx [file pone.0250014.s020.docx]

09 March 2021

Dear Editor,

**Revisions for manuscript ref: PONE-D-20-24920 “What empowerment indicators are important for food consumption for women? Evidence from 5 sub-Sahara African countries”**

Thank you for the careful appraisal and detailed comments from both editor and reviewers. We value the opportunity to revise and improve this paper. Find below our point by point response to reviewer comments. Where appropriate, changes have been made in track changes.

Reviewer #1: Thank you for addressing the comments on empirical strategy. I do not have further comments on this aspect of the paper.

However, I feel that my comments 1 (norms and country context on nutrition) and 2 (literature on empowerment and women's nutrition) have not been adequately addressed. The motivation of the paper and the literature on the pathway of linking empowerment to nutrition are missing, so the paper starts with the introduction and goes next to data description. The motivation, the nutrition framework, and how the paper contributes to the literature need greater detail. How different is this paper from the other papers on this topic? The discussion of results and conclusion are also quite generic. I think adding these sections and giving more detail would provide a stronger case for why this paper should be important.

**Response:** Many thanks for your comments. We have updated the introduction and discussion sections to include more details on the link between context and nutrition and have also made a better motivation for the study.
